# Baseline factors predicting change from the initial DMARD treatment during the first 2 years of rheumatoid arthritis: experience in the ERAN inception cohort

**DOI:** 10.1186/1471-2474-14-153

**Published:** 2013-05-01

**Authors:** Daniel F McWilliams, Patrick D W Kiely, Adam Young, David A Walsh

**Affiliations:** 1 Division of Academic Rheumatology, Arthritis Research UK Pain Centre, Clinical Sciences Building, City Hospital, Nottingham, NG5 1PB, UK; 2 St Georges Healthcare NHS Trust, London, UK; 3 West Hertfordshire Hospitals NHS Trust, St Albans, UK; 4 Sherwood Forest Hospitals NHS Foundation Trust, Sutton-in-Ashfield, UK

**Keywords:** Rheumatoid arthritis, DMARD, Sulfasalazine, Methotrexate, Cohort

## Abstract

**Background:**

Outcomes in early Rheumatoid Arthritis (RA) may be improved by rapidly establishing a stable and effective disease modifying anti-rheumatic drug (DMARD) treatment regimen. We aimed to investigate whether baseline factors and initial treatment strategies are associated with changes to the first DMARD treatment, due to either Lack of Efficacy (LoE) or Adverse Drug Reaction (ADR) within 2 years of presentation.

**Methods:**

Reasons for changes from initial DMARD therapy within 2 years of baseline, and associated factors, were examined using logistic regression in data from the Early RA Network (ERAN) inception cohort.

**Results:**

Data were available for 766 participants. 410 (54%) changed their initial DMARD regime within 2 years, including 230 (56%) due to Lack of Efficacy (LoE) and 139 (34%) due to Adverse Drug Reaction (ADR). The first DMARD was recorded as methotrexate monotherapy in 336 (44%), sulphasalazine monotherapy in 273 (36%), or combined methotrexate/sulphasalazine/hydroxychlorquine in 52 (7%).

Baseline predictors of changing DMARD (for all reasons) were HAQ-disability (aOR 1.44, 95% CI 1.12 – 1.86), poor mental health (aOR 1.44, 95% CI 1.16 – 1.78) and extra-articular disease (aOR 1.78, 95% CI 1.00 – 3.16). In this model, the triple combination therapy also predicted lower likelihood of DMARD change (aOR 0.30, 95% CI 0.12 – 0.79).

Subgroup analyses showed that MTX monotherapy was associated with lower risk of change due to ADR. Combination therapy conferred lower risk of change due to LoE. Poor mental health was associated with change due to ADR, and extra-articular disease, HAQ-disability at baseline, and younger age predicted LoE.

**Conclusions:**

Our findings suggest that non-pharmacological interventions to improve disability and mental health, may reduce initial DMARD treatment failure.

## Background

In early RA, the introduction of an effective initial therapy and rapid control of inflammatory disease activity can improve both short and long term outcomes [1-3]. Combination disease modifying anti-rheumatic drug (DMARD) therapies are recommended for initial treatment of severely active rheumatoid arthritis, although many patients continue to be treated with monotherapies [4-6]. Failure of the initial treatment, often due to inadequate benefit or adverse events, can delay treatment optimisation and disease stabilisation.

Methotrexate (MTX) is often considered the most efficacious of traditional DMARD monotherapies and may be discontinued less often than other DMARDs [2]. In studies of established RA, average durations for which MTX treatment was maintained ranged between 19 and >96 months [1,7-10]. Studies of discontinuation in early RA support these findings from established disease. Within 2 years of treatment initiation, MTX monotherapy was discontinued in 15%, compared to sulphasalazine (SSZ) in 40% of people with early RA [11], and MTX was continued on average for 60 months, compared to 21 months for SSZ [12]. MTX may be discontinued less often than other DMARDs even if previous treatments have failed [13].

Reported risk factors for the discontinuation of DMARDs are increasing age or pre-treatment anxiety at baseline [11,14], shorter disease duration, or previous failure of DMARDs [12]. Disease activity may be associated with initial DMARD choice, but is not necessarily associated with discontinuation [8]. It is unclear, however, whether these or other factors are the main predictors of DMARD discontinuation in early RA, or whether different factors predict discontinuation either due to lack of efficacy or to adverse events [11]. Better understanding factors that may contribute to discontinuation of first DMARD could aid clinicians to more rapidly establish people with early RA on their definitive treatment.

The Early Rheumatoid Arthritis Network (ERAN) is an inception cohort of people with newly diagnosed RA which commenced in 2002. We aimed to identify clinical and quality of life measures associated with early DMARD discontinuation, and discontinuation due to ADR or LoE, within 2 years of the first diagnosis.

## Methods

### Patients and recruitment

The ERAN inception cohort study, recruits from 22 outpatient centres in the UK and Eire [15]. Recruitment began in April 2002. By the end of July 2009, 1133 patients had enrolled in the study. Participants are recruited following the first diagnosis of RA by a rheumatologist and clinical data collected at baseline, 3 to 6 months, and annually from baseline thereafter. People whose diagnosis was subsequently changed were withdrawn and removed from the study database. ERAN centres treat patients according to local practice. The study was approved by Trent Research Ethics Committee (ref 01/4/047) and all participants gave signed, informed consent in line with the Declaration of Helsinki.

### Data collection

Data collected at baseline and drug data up to the 2 year follow up (visit number 4, median time = 24 months) were used for this study. At baseline clinicians recorded extra-articular disease manifestations [16] or co-morbidities [17] as previously described. Participants reported the duration of their symptoms and completed Short Form 36 (SF36) [18] and Health Assessment (HAQ) [19] questionnaires. SF36 subscales were not “normed” for age or gender (as both would be adjusted for during analyses). Erythrocyte sedimentation rate (ESR) was obtained from the clinical record. Rheumatoid factor (RF) and/or cyclic citrullinated proteins (CCP) were pooled to give either seropositive (RF positive and/or CCP positive) or seronegative (negative or weakly positive for RF and negative for CCP) classifications. The DAS28-ESR score was used as the measure of disease activity [20], and the DAS28-P score was calculated as the proportion of disease activity score contributed by patient-reported factors (tender joint count and general health visual analogue scale) [21]. Whether 4 or more of the 1987 ACR RA classification criteria were satisfied at baseline was derived from the ERAN database [22]. Baseline radiographs of hands and feet were examined by staff at the collecting ERAN centre and classified as having one or more erosions or no erosions.

### Change of initial DMARD treatment

Data were analysed from participants who had been recruited prior to July 2009, ≥ 2 years before data retrieval for this analysis, and who had commenced DMARD before visit 4. The reason for stopping or changing DMARD was recorded by each study centre as “adverse drug reaction” (ADR), “lack of efficacy” (LoE), “ADR *and* LoE”, “surgery”, “patient choice”, “non-compliance”, “other” or “unknown”. “Change” was defined as stopping all DMARD therapy for at least 2 months, altering the existing DMARD therapy (including addition of DMARDs to introduce combination therapy) or switching DMARD regimes (with one beginning < 3 months from the other finishing). Introduction of biologic therapies were recorded as changes, but alteration of DMARD dosages or route of administration were not examined in these analyses. When no reason was recorded for the addition of new DMARD therapy, it was assumed that this was due to lack of efficacy, and these patients were added to the LoE group (similarly to other reports [7]). In those patients for whom follow up data were missing between baseline and visit 4 or alteration of DMARD regime, a version of the “last observation brought forward” approach was used where those that did not change (n = 331) were excluded, and those that changed before visit 4 were included (n = 128). Those who were not prescribed DMARDs before study visit 4 (n = 8) or who had incomplete data records (n = 28) were excluded from analysis.

### Statistical analysis

Univariate data analyses were performed using Mann–Whitney U-tests and *χ*^2^ tests for categorical data, not corrected for multiple comparisons. For analysis purposes baseline DAS28 scores were classified into EULAR activity groups (0–3.19, 3.2-5.19, ≥5.2 [23]) (only 53 people were in DAS28 remission (<2.6) at baseline, and therefore remission and low disease activity subgroups were combined for analysis); BMI was classified into WHO groups (<25; 25–29.9; ≥30 [24]); and other continuous variables were divided into quartiles of increasing severity/magnitude. The highest quartile was always the most severe or least beneficial (eg. high pain, low mental health, high HAQ score etc.) and the least severe was used as the reference. Logistic regression was used to examine independent associations between baseline factors and changing of DMARDs within 2 years, followed by risks for changing due to LoE and ADR. Categorical dummy variables for initial DMARDs were limited to MTX monotherapy, SSZ monotherapy and triple therapy due to clinical relevance and low usage of other therapies. Logistic regression was performed to investigate independent risk factors for DMARD change. Age, gender, DAS28 and baseline DMARD regimen were all included in each model, as key descriptive clinical variables. Other variables were selected if associated with DMARD change at the univariate level for one of the analyses. The same variables were included in each logistic regression model for each outcome. Sensitivity analyses were also performed using different definitions of combination therapy, smoking history and diagnostic criteria. Statistical significance was taken when p < 0.05.

## Results

**Figure 1:**
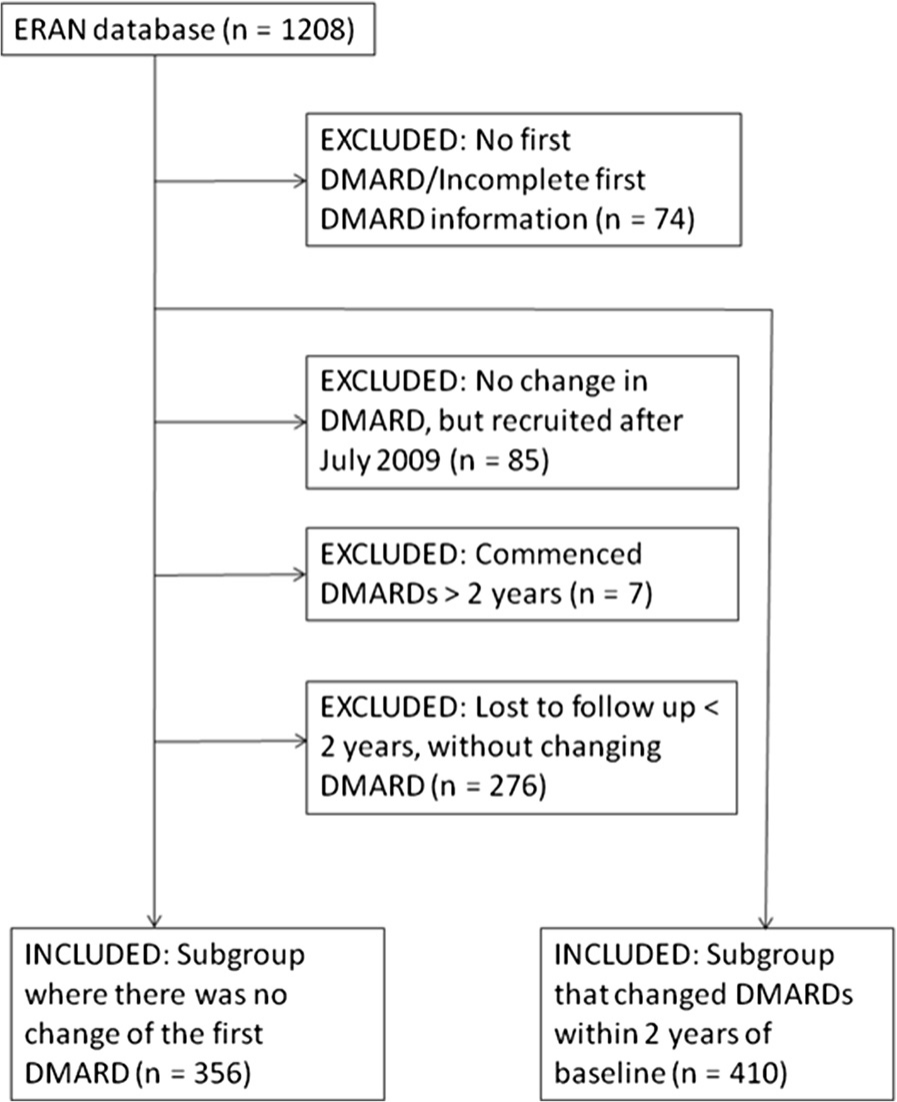
Selection of participants for analysis.

A total of 766 people had data available for analysis. Reasons for inclusion/exclusion are summarised in Figure 1. Age and gender prevalence were similar for included and excluded cases, but those included in the analyses had, at baseline, slightly higher DAS28 scores (median (IQR) 5.8 (4.6 – 7.0) vs 5.5 (4.1 – 6.7)) and slightly greater HAQ-disability (median (IQR) 1.1 (0.6 – 1.8) vs 0.9 (0.4 – 1.5)).

Of the 766 participants, 54% (n = 410) changed their initial DMARD regime between baseline and study visit 4. This group was comprised of those where an additional DMARD had been added (n = 182), those that switched to a new DMARD (n = 134) and those where DMARD treatment ceased for at least 2 months (n = 94). The commonest reasons for changing a DMARD were ADR (139 (34%)) and LoE (230 (56%)). “Other” was recorded for 22 patients and other codes were assigned to the remaining 19 patients (including the code for LoE & ADR combined). The comparator group (n = 356) had not changed their initial DMARD by study visit 4 (median time from baseline to study visit 4 was 24 months).

The initial DMARD treatments are listed in Table 1 and the patient demographics are shown in Table 2. Participants given initial SSZ monotherapy had slightly better median (IQR) DAS28 (4.7 (3.7 – 5.7)) and were less likely to be seropositive (52%) than those initially treated with MTX monotherapy (DAS28: 5.1 (4.1 – 6.1) and 66% seropositive; each p < 0.05). Otherwise, the demographics of three commonest treatment groups were statistically similar.

**Table 1:** Initial DMARD regimens

**Initial DMARD regimen**	
Monotherapies (n = 670)	
MTX	43.9%
SSZ	35.6%
HCQ	5.4%
Lef	1.6%
Others	1.0%
Combination therapies (n = 96)	
MTX + SSZ + HCQ	6.8%
MTX + SSZ	2.7%
MTX + HCQ	1.4%
Others	1.6%

Prevalences of intial DMARD treatments in ERAN participants stratified into monotherapies and combination therapies. *MTX* methotrexate, *SSZ* suplphasalazine, *HCQ* hydroxychloroquine, *Lef* leflunomide.

**Table 2:** Characteristics of patient study groups

	**No change of DMARD**	**Change of DMARD**	**Reason for change**	
			**Adverse drug reaction**	**Lack of efficacy**
**N**	356	410	139	230
**Female**	65%	72% *	71%	72%
**Age (yrs)**	58 (47–68)	56 (46–66)	60 (50–70)	54 (44–63) **
**BMI (kg/m2)**	26.4 (23.9 - 30.1)	26.9 (23.7 - 30.8)	26.6 (23.8 - 30.5)	27.2 (23.9 - 31.2)
**Ever smoked**	57%	65% *	66%	65%
**Current smoker**	35%	39%	39%	37%
**Symptom duration (mo)**	6 (4–12)	6 (4–12)	5 (3–10)	6 (3–13)
**Erosions**	47%	26%	23%	28%
**HAQ (0–3)**	1.0 (0.4 - 0.6)	1.4 (0.8 - 1.9) **	1.3 (0.6 - 1.8) *	1.4 (0.8 - 1.9) **
**>4 of 1987 ACR criteria**	51%	59% *	53%	65% **
**Seropositive**	62%	61%	54%	64%
**Extra-articular disease**	11%	20% **	15%	24% **
**Co-morbidity**	44%	50%	56% *	49%
**DAS28**	4.6 (3.6 - 5.7)	5.1 (4.0 - 6.1) **	4.8 (3.5 - 5.7)	5.4 (4.1 - 6.2) **
**DAS28-P (0–1)**	0.41 (0.32 - 0.47)	0.43 (0.35 - 0.50) **	0.42 (0.32 - 0.50)	0.43 (0.37 - 0.50) **
**SF36-Bodily pain (0–100)**	41 (31–62)	32 (22–51) **	41 (22–62) *	31 (21–42) **
**SF36-Mental Health (0–100)**	68 (52–80)	60 (48–72) **	60 (48–72) **	56 (48–70) **
**Steroid at baseline**	17%	16%	14%	16%
**Sulphasalazine monotherapy**	27%	43%	59%	33%
**Methotrexate monotherapy**	53%	36%	23%	48%
**Other monotherapy**	4%	11%	9%	12%
**Dual therapy**	6%	5%	7%	4%
**Triple therapy**	10%	4%	2%	4%

Comparison of patient groups based upon DMARD changes. Medians (IQR) or percentage prevalences are presented. ** p < 0.01, * p < 0.05 for comparison vs No Change. Statistically significant results are presented in **bold** (with the exception of DMARD treatments, which were also significantly heterogeneous, but tested as a group).

Table 2 shows the univariate analyses of baseline factors compared between groups. Univariate analysis found that the recorded reasons for changing DMARDs differed between groups (change for any reason *χ*^2^ = 31.2, p < 0.001; LoE *χ*^2^ = 10.7, p = 0.014, ADR *χ*^2^ = 63.9, p <0.001). Changing DMARD was associated with gender, previous smoking, HAQ disability, 1987 ACR criteria, extra-articular disease, DAS28, DAS28-P, Bodily Pain and Mental Health scores. There were 111/336 (33%) patients reported as changing from MTX monotherapy due to LoE and 27/336 (8%) changed due to ADR. With SSZ monotherapy, 76/273 (28%) changed due to LoE and 85/273 (31%) due to ADR. One hundred and twenty two people (16%) were receiving steroids at baseline, but this was not significantly associated with the future change of DMARD treatment (Table 2). Neither MTX monotherapy, SSZ monotherapy nor triple therapy were more likely to be associated with baseline steroid usage (data not shown). In those that changed their initial DMARD treatment, the median (IQR) times until change were 6 (3 – 11) months for SSZ, 9 (6 – 16) months for MTX and 17 (8 – 24) for triple therapy.

Logistic regression was used to examine baseline factors associated with subsequent change of DMARD for any reason, including both LoE and ADR (Table 3). Increasing HAQ-disability (aOR 1.44 (95% CI 1.12 - 1.86, p = 0.005); extra-articular disease (aOR 1.78, 95% CI 1.00 – 3.16, p = 0.050); and worse SF36-Mental Health subscale scores (aOR 1.44, 95% CI 1.16 – 1.78, p < 0.001) were each associated with change of DMARD. Triple therapy as first treatment was associated with less risk of subsequent DMARD change (aOR 0.30, 95% CI 0.12 – 0.79, p = 0.014). Head to head comparative analysis between MTX monotherapy and SSZ monotherapy favoured MTX as initial DMARD (aOR 0.41, 95% CI 0.28 – 0.60, p < 0.001). Sensitivity analyses showed that exchanging previous smokers for current smokers did not alter the main findings, and did not yield a statistically significant association between smoking and DMARD change. Findings also were unchanged by only including people that commenced DMARD therapy within 12 months from baseline, instead of including all people who commenced DMARDs before visit 4 (data not shown). Furthermore, sensitivity analyses only including participants who satisfied ACR 1987 classification criteria for RA did not affect statistical associations between baseline factors and DMARD change (data not shown).

**Table 3:** Logistic regression models of non-persistence of DMARD

**Variable**	**Categories**	**Risk of change**	**Reasons for changing DMARD**	
			**Risk of adverse drug reaction**	**Risk of lack of efficacy**
**Female gender**	Y/N	1.11 (0.69 - 1.80)	1.52 (0.81 – 2.85)	1.20 (0.71 – 2.03)
**Age**	Quartiles	0.89 (0.72 - 1.10)	1.23 (0.94 – 1.60)	0.69 (0.55 – 0.87) **
**Ever smoked**	Y/N	1.38 (0.89 - 2.15)	1.29 (0.59 – 2.78)	1.35 (0.83 – 2.19)
**DAS28**	EULAR categories	1.12 (0.78 - 1.62)	0.95 (0.58 – 1.57)	1.29 (0.83 – 2.02)
**>4 of 1987 ACR criteria**	Y/N	0.98 (0.59 – 1.65)	1.04 (0.57 – 1.91)	1.20 (0.72 – 2.01)
**Extra-articular disease**	Y/N	1.78 (1.00 - 3.16) *	1.29 (0.59 – 2.78)	2.27 (1.25 – 4.11) **
**Co-morbidities**	Y/N	1.02 (0.66 - 1.57)	1.18 (0.67 – 2.09)	0.82 (0.51 – 1.32)
**HAQ score**	Quartiles	1.44 (1.12 - 1.86) **	1.10 (0.80 – 1.52)	1.36 (1.03 – 1.81) *
**SF36-bodily pain**	Quartiles	0.93 (0.72 - 1.19)	1.03 (0.75 – 1.43)	1.18 (0.90 – 1.55)
**SF36-mental health**	Quartiles	1.44 (1.16 - 1.78) **	1.43 (1.08 – 1.89) *	1.22 (0.97 – 1.52)
**DAS28-P**	Quartiles	1.09 (0.88 - 1.35)	1.04 (0.79 – 1.37)	1.10 (0.86 – 1.41)
**SSZ monotherapy**	Y/N	1.09 (0.57 - 2.12)	1.92 (0.85 – 4.37)	0.74 (0.35 – 1.54)
**MTX monotherapy**	Y/N	0.56 (0.29 - 1.06)	0.38 (0.16 – 0.94) *	0.57 (0.28 – 1.17)
**MTX + SSZ + HCQ**	Y/N	0.30 (0.12 - 0.79) *	0.33 (0.08 – 1.38)	0.14 (0.04 – 0.53) **

Variables associated with a non-persistence of DMARD treatment within the first 2 years in the ERAN cohort (428 people had data analysed). Adjusted odds ratios (95% confidence intervals). * = p < 0.05, ** = p < 0.01. Statistically significant results are presented in **bold**.

Analyses of subgroups were performed for change due to LoE or ADR. Logistic regression models showed that change due to LoE was positively associated with extra-articular disease and with HAQ-disability, and negatively associated with age and triple therapy as initial DMARD regime. ADR was associated with worse SF36-Mental Health subscale scores and negatively with MTX monotherapy (Table 2).

In further sensitivity analyses, substituting all MTX-containing combinations instead of MTX + SSZ + HCQ triple therapy did not affect the significant findings. For example, in logistic regression for DMARD change for all reasons, the statistically significant aOR (95% CI) were Mental Health score 1.32 (1.08 – 1.60), p = 0.006; extra-articular disease 1.81 (1.06 – 3.07), p = 0.028; HAQ 1.32 (1.05 – 1.67), p = 0.020; MTX monotherapy 0.25 (0.12 – 0.54), p < 0.001; MTX-combination 0.17 (0.07 – 0.43), p < 0.001).

## Discussion

In this study we found that changing from the first prescribed DMARD within 2 years of presentation was predicted by the baseline higher levels of HAQ-disability, worse mental health and presence of extra-articular disease. Also, the choice of first DMARD was associated with the risk of subsequent change, with patients most likely to persist with MTX (consistent with a previous report [12]) and triple therapy of MTX + SSZ + hydroxychloroquine.

We and others have shown previously that choice of SSZ or MTX as initial DMARD in patients with early rheumatoid arthritis was associated with classical prognostic factors and disease activity, particularly rheumatoid factor seropositivity and swollen joint counts [6,25]. Extra-articular disease at baseline, another poor prognostic factor, was associated with DMARD changes due to LoE, and others have found that extra-articular disease may predict adverse events on DMARDs [26].

Contemporaneous disease activity influenced decisions to change from the initial DMARD, as indicated by the large proportion of patients whose change was attributed to LoE. However, LoE during the first 2 years was not predicted by baseline disease activity, and we found that factors other than inflammatory disease prognosis were important predictors of DMARD change. The effects of mental health and disability in predicting DMARD change were independent of RA inflammatory disease severity as measured by DAS28, which was adjusted for in the logistic regression analyses. Pain at baseline significantly predicted DMARD change at the univariate level, although significance was lost after adjustments for other factors. These findings suggest that in contemporary practice which focuses on suppression of inflammatory disease activity, other symptoms such as poorer mental health or higher HAQ-disability at baseline may affect DMARD discontinuation decisions in the clinic, regardless of the measurable disease activity or reported pain.

We found that poorer mental health scores at baseline predicted subsequent change in DMARD regimen, especially for ADR. Listing et al., 1997 reported that poor mental health was associated with subsequent DMARD discontinuation in people with early RA [11], and people with higher levels of anxiety were more likely to discontinue initial DMARD [14]. Poor mental health may be associated with non-inflammatory pain mechanisms in RA, and approximately 20% of people with RA may fulfill fibromyalgia classification criteria [27]. Influence of mental health status and non-inflammatory pain mechanisms on DMARD discontinuation deserves further study. Psychological and medical interventions have potential to improve mental health, and therefore facilitate continuation with DMARD regimens, especially if poorer mental health is associated with poorer treatment adherence. The prediction of DMARD changes by baseline disability indicates that initial DMARD monotherapy may deal inadequately with disability. Further studies would be required to determine whether addressing disability itself may facilitate DMARD continuation.

As expected, the original DMARD choice also predicted changes to DMARDs during the first 2 years. Use of MTX alone or as a part of combination therapy was associated with lower risk of DMARD change, either due to LoE or ADR. Current UK guidelines recommend that the initial treatment of active RA should include MTX [28]. Our data indicate that the greater likelihood that regimens containing MTX will be continued [7,29] is independent of baseline disease activity. This suggests that MTX should not be withheld from patients with low or moderate disease activity at baseline.

We were unable to confirm previous findings that older patients were more likely to discontinue DMARDs [14]. Indeed, older participants were less likely to change DMARD due to LoE, possibly indicating a greater acceptance of disease activity by patients or their physicians. Consistently with this, older patients in ERAN were less likely to be prescribed DMARDs. However, further research is required to determine whether different treatment strategies should be adopted in elderly patients.

There are several limitations to this analysis. We used DAS-ESR a validated measure of inflammatory disease activity in common clinical use. However, DAS-ESR may be affected by factors other than disease activity [21] and further work would be required to determine whether other baseline measures of disease activity, for example incorporating C-reactive protein rather than ESR, may predict subsequent DMARD changes. DMARDs were sometimes changed without the reason being formally recorded, although it is likely that addition of a new DMARD whilst continuing the initial DMARD represents lack of adequate efficacy of the initial monotherapy [7]. Also, the clinical decision-making process is complex, taking account of patient and physician preferences, changing local guidance as well as LoE and ADR. Our subgroups may represent heterogeneous reasons for DMARD change that could not be fully elucidated. Not all DMARD treatments could be modelled without over-adjusting the results. The two commonest monotherapies and triple DMARD therapy were included as being most-relevant to clinical practice, and further research may determine whether similar factors predict discontinuation of other initial DMARD regimens.

## Conclusions

This is a large study of DMARD change in early RA. Changing within 2 years from initial DMARD therapy was predicted by baseline disability, poorer mental health and the presence of extra-articular disease. Early initiation of intensive treatment, including MTX at baseline, and non-pharmacological measures to improve disability and mental health may have potential to reduce morbidity and cost from initial treatment failure.

## Competing interests

This study was funded by a grant from Pfizer Ltd (grant number WS953552) which also funded D McWilliams. D Walsh is the grant holder. A Young and P Kiely declare no competing interests.

## Authors’ contributions

DM designed and performed the data analysis. PK conceived of the study and aided in the design of the study. AY conceived of the study, aided in the design of the study, retrieved the data from the ERAN database and supervised the administration/coordination of the ERAN cohort. DW conceived of the study and designed the data analysis. All authors drafted the manuscript, critically read the drafts and suggested improvements and approved the final version.

## Authors’ information

Daniel McWilliams and David Walsh are guarantors for the data and analysis performed in this study.

## Pre-publication history

The pre-publication history for this paper can be accessed here:

http://www.biomedcentral.com/1471-2474/14/153/prepub
